# Bridging smiles: AI-driven dental health awareness for the next gen—a systematic review

**DOI:** 10.3389/fdmed.2025.1639572

**Published:** 2025-10-17

**Authors:** Pratyasha Sharma, Sayan Das, Srikala Bhandary, Prajna P. Nayak

**Affiliations:** Department of Paediatric & Preventive Dentistry, AB Shetty Memorial Institute of Dental Sciences (ABSMIDS), Nitte (Deemed to be University), Mangalore, Karnataka, India

**Keywords:** oral health, children, game-based teaching, serious games, interactive games, education

## Abstract

**Background:**

This systematic review aims to evaluate the effectiveness of mobile applications in improving oral health knowledge, oral hygiene behaviours, plaque, and gingival indices in children.

**Methods:**

The inclusion criteria were defined using the PICOS framework. The population (P) comprised children aged 4–16 years. The intervention (I) included studies utilizing game-based teaching methods for oral health education, such as serious games, interactive games, or other digital and non-digital educational games. Comparators (C) involved alternative interventions, conventional teaching approaches, or control conditions. Eligible outcomes (O) included measures of oral hygiene behaviours, plaque and gingival indices. 5 Randomized controlled trials, 3 pilot studies and 1 cross-sectional study were considered for inclusion after going through a total of 163 articles. Reviews and usability test articles were excluded from the study. A systematic search was completed using keywords alongside thesaurus and MeSH terms on PubMed, Scopus, Cochrane Library and Web of Science databases from 2015 to 2025. This review was conducted by the Preferred Reporting Items for Systematic Reviews and Meta-Analyses (PRISMA) checklist, using the specified inclusion and exclusion criteria. The Risk of Bias was assessed using the QUADAS-2 tool. All results were analyzed and summarized into general and specific categories.

**Results:**

Nine randomized controlled trials involving children aged 2–19 years were included. Mobile applications used ranged from brushing timers and virtual games to educational videos and messaging platforms. Most interventions reported improvements in oral health knowledge, hygiene behaviours, and plaque reduction. Significant clinical improvements were observed in several studies, particularly those incorporating gamification or theory-based behavioural strategies.

**Discussion:**

Mobile-based interventions showed promising results in enhancing oral health outcomes among children. Gamified and interactive applications were especially effective in increasing motivation and engagement. However, findings varied across studies, with some reporting comparable or superior outcomes in control groups receiving conventional education. Parental involvement and age-specific tailoring appeared to influence intervention effectiveness.

**Conclusion:**

Mobile health applications have the potential to support pediatric oral health promotion through improved knowledge, behaviour, and clinical outcomes. While the evidence is encouraging, further standardized, high-quality research is needed to confirm long-term effectiveness and guide widespread implementation.

**Systematic Review Registration:**

https://www.crd.york.ac.uk/PROSPERO/search, PROSPERO CRD420251065550.

## Introduction

Artificial Intelligence (AI) is transforming dental health awareness by offering innovative and tailored solutions for future generations. As smartphones and tablets become more prevalent among young people, mobile apps have become cutting-edge tools for educating about oral health. AI-driven chatbots, mobile apps, and virtual assistants are making dental health information more accessible to children, teenagers, and young adults. By incorporating gamification, animation, and augmented reality (AR), these tools encourage good oral hygiene practices and create a user-friendly experience, enhancing knowledge retention in children and fostering positive attitudes towards oral health. This review explores various studies that evaluate different app-based interventions, assessing their effectiveness, advantages, and challenges in promoting healthy oral habits among children.

Oral health is a fundamental component of overall well-being, particularly in childhood, when lifelong health behaviours are established. Poor oral health in children can lead to pain, infection, impaired nutrition, speech difficulties, and a diminished quality of life. Globally, over 600 million children are affected by oral health problems, highlighting the urgent need for effective preventive and educational strategies (1).

With the global rise in smartphone ownership, estimated at 3.5 billion users in 2020 and increasing in the last 5 years, there is a higher potential for leveraging mobile technologies to support health promotion efforts, particularly in developing regions such as China, India, and Latin America, where smartphone use is spreading rapidly (2).

Health education plays a pivotal role in promoting oral hygiene and preventing dental diseases. Research indicates that children as young as six years old often demonstrate greater digital literacy than adults (3). Leveraging digital platforms as part of a behavioural theory-based strategy offers a promising avenue for long-term improvements in oral health. Smartphone applications, in particular, can serve as vehicles for delivering oral health education, reinforcing behaviours such as proper toothbrushing and dietary habits (4). Among these, game-based interventions have emerged as a promising approach, leveraging elements of play to improve motivation and learning outcomes in health-related behaviours.

Numerous patient-focused mobile applications are already in use, demonstrating promise in various health domains. For example, the use of text message reminders (SMS) has been shown to significantly enhance oral hygiene behaviours among young adults aged 18–24, with rates of regular toothbrushing increasing from 51% to 73% over a 12-week intervention period (5). Similarly, mobile applications that offer brushing timers and customizable reminders have proven effective in encouraging users to maintain optimal oral hygiene routines, such as brushing for two minutes and replacing toothbrushes regularly (6).

Despite the growing interest in digital health interventions, the evidence surrounding the effectiveness of game-based applications in improving children's oral health remains scattered. Furthermore, few systematic reviews have focused specifically on mobile or game-based educational tools in this context. Therefore, this systematic review aims to evaluate the effectiveness of game-based teaching methods delivered through mobile or digital platforms in enhancing oral health knowledge, improving hygiene behaviours, and reducing plaque accumulation among children aged 4–16 years.

## Materials and methods

The present review was conducted according to the Preferred Reporting Items for Systematic Reviews and Meta-Analyses (PRISMA) checklist (7) to ensure the inclusion of relevant studies.

### Literature search

Multiple electronic databases, including PubMed, Web of Science, Scopus, Cochrane Library were searched using a combination of keywords and Medical Subject Headings (MeSH) terms, from 2015 to 2025.

The search strategy combined terms related to oral health, children, game-based teaching, serious games, interactive games and education. Boolean operators (AND, OR) were used to combine the search terms effectively. No filters or date restrictions were placed with only English language publications considered for inclusion.

To ensure the inclusiveness of the search, additional sources were explored, including conference proceedings, and grey literature databases.

### Inclusion and exclusion criteria for study selection

The inclusion criteria for selecting studies in the PICOS frame work was:

Population (P): studies involving children aged 4–16 years were considered.
Intervention (I): studies that employed game-based teaching interventions for oral health education were included. This encompassed serious games, interactive games or other forms of digital or non-digital games used as an educational tool.
Comparison (C): studies that examined the effectiveness of game-based teaching compared to alternative interventions, conventional teaching methods or control conditions.
Outcome (O): studies assessing oral health-related outcomes such as
  (a) Oral hygiene behaviours
  (b) Plaque scores.
  (c) Gingival index
Study Design (S): randomized controlled trials, pilot studies, cross-sectional studies.

### Data extraction phase

Two reviewers (PS and SD) independently assessed the search results to identify relevant articles. The reviewers were not blinded to the author names or affiliations. Non-relevant articles and duplicates were excluded. In the initial screening, titles and abstracts were reviewed to determine their relevance to the research question and the predefined inclusion criteria. The full texts of potentially relevant articles were then assessed in the second screening phase to determine their eligibility for final inclusion. The reference lists of relevant articles were manually searched for any additional studies that may be included. Discrepancies between reviewers were resolved by a third author (SB) and fourth author (PN) through consensus. The search strategy is shown in Table 1.

**Table 1:** Search strategy and queries used for the review.

Database	Search query	Results
PubMed	((((((((((((((((Mobile gaming) OR (Mobile game)) OR (Game)) OR (Smartphone app)) OR (Smartphone application)) OR (Smartphone game)) AND (Oral health)) OR (Oral hygiene)) OR (Oral education)) OR (Toothbrushing)) OR (Dental hygiene)) OR (Dental health)) OR (Dental)) AND (Children)) OR (Child)) OR (Pediatric)) OR (Pediatric Dentistry)	110
Scopus	TITLE-ABS-KEY ((((((((((((((((Mobile gaming) OR (Mobile game)) OR (Game)) OR (Smartphone app)) OR (Smartphone application)) OR (Smartphone game)) AND (Oral health)) OR (Oral hygiene)) OR (Oral education)) OR (Toothbrushing)) OR (Dental hygiene)) OR (Dental health)) OR (Dental)) AND (Children)) OR (Child)) OR (Pediatric)) OR (Pediatric Dentistry)	23
Web of science	All Fields ((((((((((((((((Mobile gaming) OR (Mobile game)) OR (Game)) OR (Smartphone app)) OR (Smartphone application)) OR (Smartphone game)) AND (Oral health)) OR (Oral hygiene)) OR (Oral education)) OR (Toothbrushing)) OR (Dental hygiene)) OR (Dental health)) OR (Dental)) AND (Children)) OR (Child)) OR (Pediatric)) OR (Pediatric Dentistry)	8
Cochrane library	((((((((((((((((Mobile gaming) OR (Mobile game)) OR (Game)) OR (Smartphone app)) OR (Smartphone application)) OR (Smartphone game)) AND (Oral health)) OR (Oral hygiene)) OR (Oral education)) OR (Toothbrushing)) OR (Dental hygiene)) OR (Dental health)) OR (Dental)) AND (Children)) OR (Child)) OR (Pediatric)) OR (Pediatric Dentistry)	11

## Results

**Figure 1:**
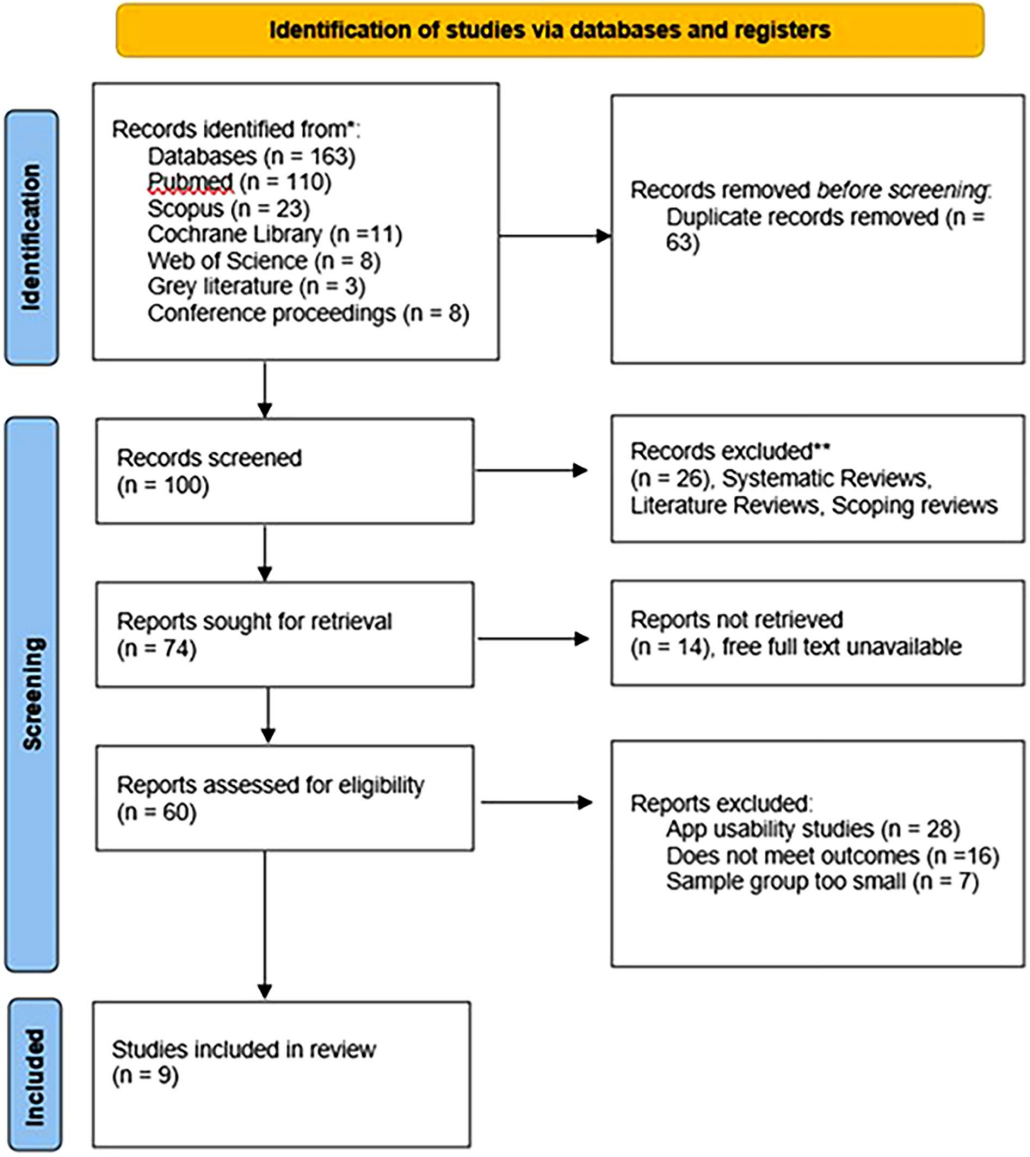
PRISMA flow diagram for the review.

As a result of database searching, 74 articles were retrieved. Next, 60 articles met the inclusion criteria and were selected for full text review. Lastly, 9 studies remained relevant articles. The procedure of screening articles based on the PRISMA method is displayed in Figure 1.

Full text of all 9 articles was reread to determine whether they met the study outcomes, and differences were tabulated.

### Risk of bias assessment

**Figure 2:**
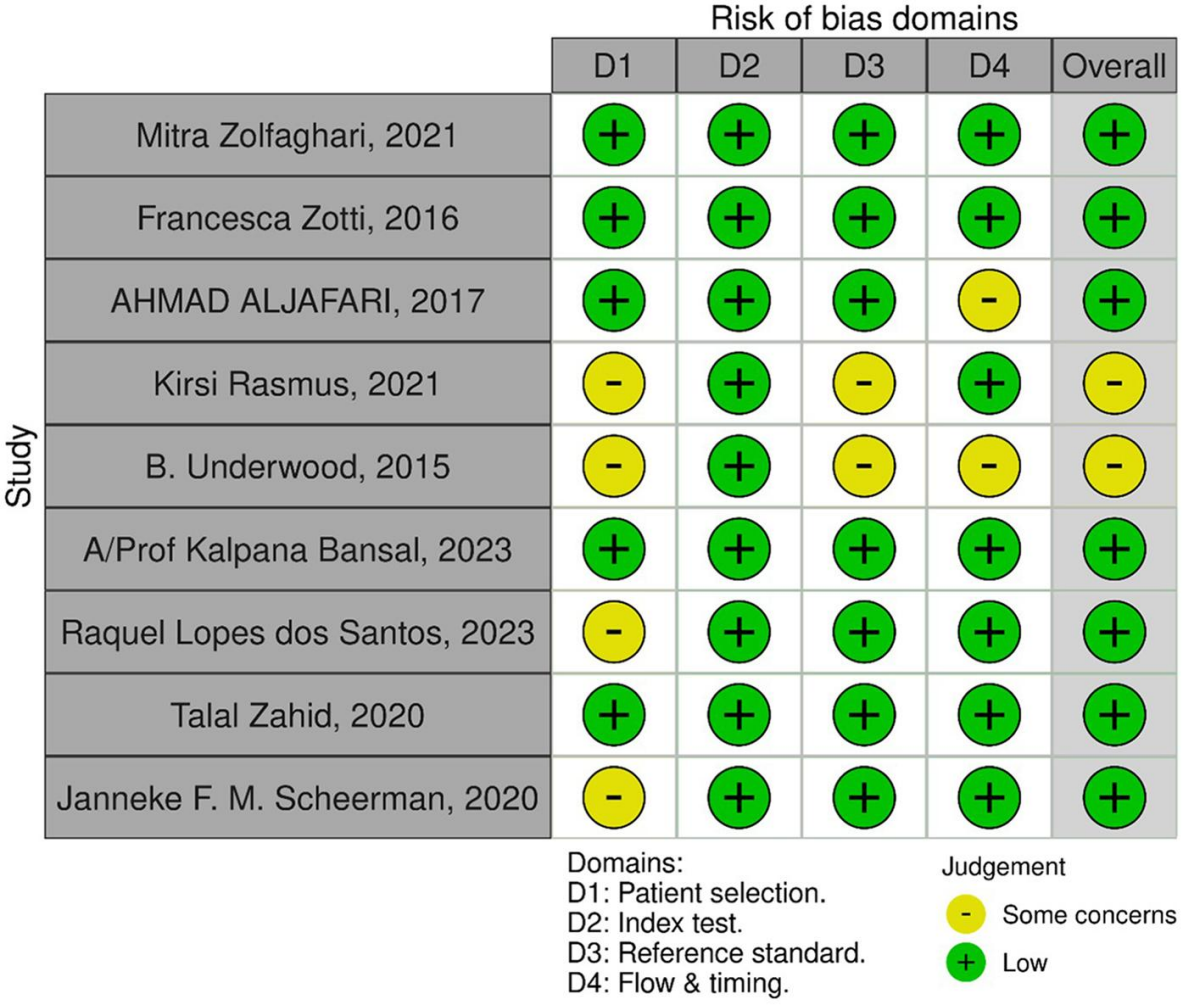
Risk of bias assessment plotted with the Robvis tool.

QUADAS-2 tool (8) was used to assess the risk of bias of each article and the results were tabulated. The Robvis tool (9) was used to generate visual plot points with the results, as displayed in Figure 2.

### Characteristics of included studies

Five articles used randomized controlled trial as their study design (10–14). Three pilot studies checked the feasibility and initial response of children and caregivers to the mobile applications (15–17). One study employed a questionnaire-based study (18).

**Figure 3:**
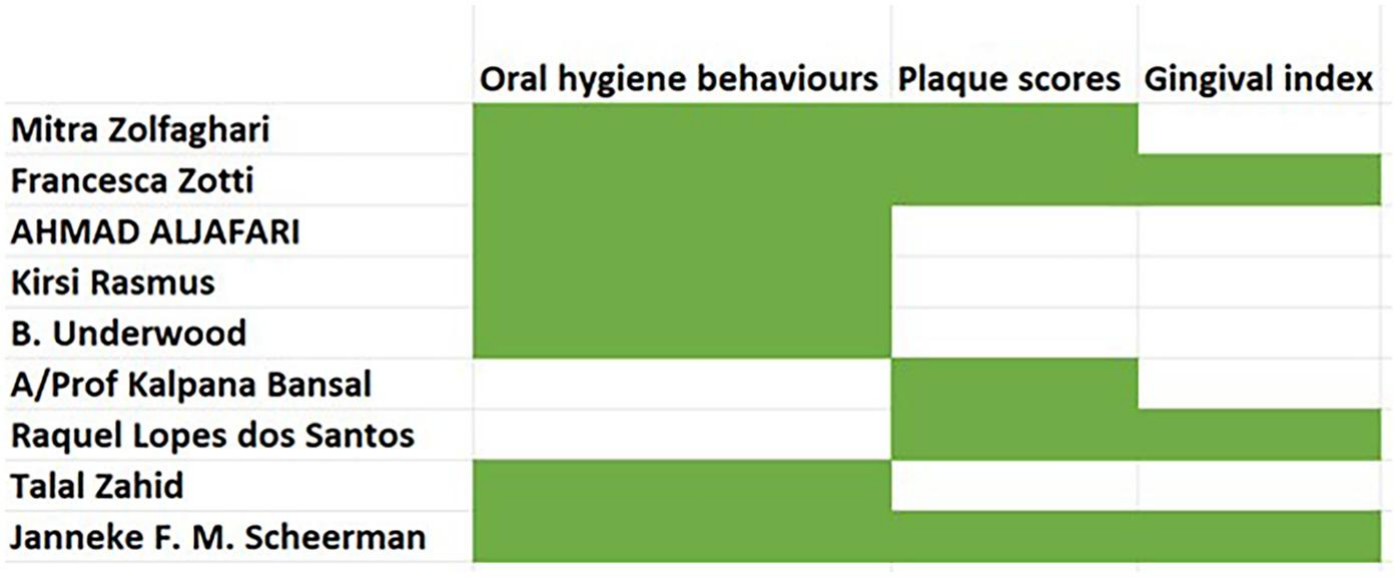
Distribution of outcomes across the studies.

The games were developed for the following applications: toothbrushing skills, reduced plaque accumulation and improved gingival health. Among the included studies, seven studies assessed the skill in oral hygiene behaviours such as toothbrushing (10–12, 14, 16, 18). Five studies assessed the change in plaque scores before and after intervention (10, 11, 13, 14, 17). Lastly, three studies further evaluated the oral health status by assessment of the gingival bleeding index (11, 14, 17). The distribution of outcomes across the studies is depicted in Figure 3.

All the studies included pediatric population (age 4–14 years), however, the age groups considered in each study varied. For example, three studies (11, 17, 18) focused primarily on the adolescent age group (11–19 years), while three studies also included the caregivers in their study group (10, 12, 16).

Tables 2, 3 depict the descriptions of the included studies.

**Table 2:** Description of mobile application used in each study.

Author, year	Age group (years)	Sample size	Mobile application intervention
B. Underwood, 2015	5–14	189	“Brush DJ” app
A/Prof Kalpana Bansal, 2023	2–10	52	“Healthy- Smile Swasth-Muskaan” app
Mitra Zolfaghari, 2021	3–7	58	Simple reminder app vs. Gamified app
Francesca Zotti, 2016	11–14	80	WhatsApp chat room “Brush Game”
Ahmad Aljafari, 2017	4–10	109	Game vs. One-on-one health education
Kirsi Rasmus, 2021	4–12	36	“Denny- The Tooth” virtual pet game and “Denny Timer” virtual toothbrushing assistant
Talal Zahid, 2020	14–16	271	“Brush DJ” app vs. Eduactional Lecture
Janneke F. M. Scheerman, 2020	12–16	132	“WhiteTeeth” app vs. usual care
Raquel Lopes dos Santos, 2023	14–19	8	“A Dentista Cientista” app vs. standard education

**Table 3:** The assessment method and outcome reported in each study.

Author, year	Assessment method	Outcomes
B. Underwood, 2015	Questionnaire about toothbrushing skills	70% participants said their teeth felt cleaner. 88% participants were motivated to brush their teeth for longer time.
A/Prof Kalpana Bansal, 2023	Mean plaque scores at one month follow up	1.6 ± 0.32–0.94 ± 0.45 (score improved after one month)
Mitra Zolfaghari, 2021	Mean plaque scores at one month follow up	Simple reminder app: Pre-intervention = 0.8, post-intervention = 0.5 Gamified app: Pre-intervention = 1, post-intervention = 0.5
Francesca Zotti, 2016	Mean plaque scores and gingival index at 3 month-intervals for 1 year	**Plaque Scores** Control group: t_0_ = 0.48, t_1_ = 1.72, t_2_ = 1.80, t_3_ = 1.85, t_4_ = 1.79 Study group: t_0_ = 0.41, t_1_ = 1.68, t_2_ = 1.45, t_3_ = 1.32, t_4_ = 1.06 **Gingival Index** Control group: t_0_ = 1.17, t_1_ = 1.35, t_2_ = 1.31, t_3_ = 1.38, t_4_ = 1.40 Study group: t_0_ = 1.18, t_1_ = 1.11, t_2_ = 0.99, t_3_ = 0.87, t_4_ = 0.67
Ahmad Aljafari, 2017	Toothbrushing frequency self-reported in a diary.	No statistical significance in toothbrushing frequency. Both groups showed adherence to brushing twice daily.
Kirsi Rasmus, 2021	Questionnaire about change in oral health routines after 5 weeks	69.4% children learnt how to brush better. 61.1% reported that brushing became a more fun activity, while 55% reported that they brushed for a full 2 min. 50% were more motivated to initiate brushing, while 36.1% reported that the results lasted beyond use of the app.
Talal Zahid, 2020	Questionnaire about oral health attitudes and behaviours	Control group showed better results in toothbrushing frequency (75% vs. 66.7% in study group) Slight improvement is seen in brushing technique in the study group (72.8% vs. 72.5% in control group)
Janneke F. M. Scheerman, 2020	Questionnaire about oral health behaviours, mean plaque scores and gingival bleeding index	At6-weekfollow-up, the intervention led to a significant decrease in gingival bleeding (B = −3.74; 95% CI−6.84 to −0.65) and an increase in the use of fluoride mouthrinse (B = 1.93; 95% CI 0.36–3.50). At 12-week follow-up, dental plaque accumulation (B = −11.32; 95% CI−20.57 to −2.07) and the number of sites covered with plaque (B = −6.77; 95% CI−11.67 to −1.87) had been reduced significantly more in the intervention group than in the control group.
Raquel Lopes dos Santos, 2023	Mean plaque scores and gingival bleeding index at baseline, 30, 60 and 90 days	Although no significant difference could be observed, plaque scores at T1 and T2 were lower in the experimental group (33.20 ± 19.29; 32.10 ± 7.72) than in the control group (42.11 ± 8.56; 43.59 ± 34.71). The same was observed for gingival bleeding index, in the experimental group presented lower GBI at T1 and T2 (12.70 ± 8.10; 13.72 ± 7.39) than in the control group (27.53 ± 17.89; 20.38 ± 9.95).

## Discussion

This systematic review aimed to evaluate the effectiveness of mobile-based applications in promoting oral health among children and adolescents, with particular emphasis on oral health knowledge, hygiene behaviours, plaque scores, and gingival health. The included studies demonstrated considerable variability in age groups, intervention types, outcome measures, and results. Despite these differences, several common themes emerged, underscoring the potential of mobile health technologies to enhance pediatric oral health education and behavioural outcomes.

A number of studies reported improvements in oral hygiene behaviours and knowledge following the use of game-based or interactive mobile applications. For example, Underwood (16) found that the *Brush DJ* app improved users' perception of tooth cleanliness and brushing duration, with 88% of participants reporting increased motivation. Similarly, Rasmus et al. (15) showed that virtual pet games like *Denny-The Tooth* not only helped children learn proper brushing techniques but also enhanced motivation and engagement in oral hygiene practices. This is consistent with previous literature on gamification in health promotion, where interactive, reward-based strategies have been shown to increase intrinsic motivation and sustain health behaviours in pediatric populations (19). The capacity of mobile-based interventions to transform routine practices like toothbrushing into enjoyable activities explains their success, particularly in the younger age group (20).

Regarding clinical outcomes, several studies demonstrated reductions in plaque scores following mobile app interventions. Bansal et al. (13) and Zolfaghari et al. (10) both reported significant decreases in plaque accumulation after one month of using mobile applications, with gamified interventions showing slightly better results than simple reminder-based apps. Zotti et al. (11) extended these findings over a one-year period, reporting sustained reductions in both plaque scores and gingival indices in the intervention group using the *Brush Game* via WhatsApp. These findings highlight that digital interventions, when designed with sustained reinforcement and interactive features, can achieve outcomes comparable to traditional supervised oral health programs. Notably, interventions that incorporated theoretical behaviour change models, such as the Health Action Process Approach or Social Cognitive Theory, showed greater efficacy (21). This suggests that mobile applications should be theory-driven rather than solely technology-driven.

Moreover, Scheerman et al. (14) provided robust evidence supporting the efficacy of the *WhiteTeeth* app. Their randomized controlled trial demonstrated significant reductions in plaque accumulation and gingival bleeding, as well as increased use of fluoride mouthrinse in the intervention group at both 6- and 12-week follow-ups. This aligns with the broader literature suggesting that behaviour change strategies grounded in theoretical models,such as self-efficacy and reinforcement,can significantly impact oral health outcomes. Similar behaviourally grounded mobile interventions have proven effective in other domains of adolescent health, such as nutrition and physical activity, underscoring the cross-disciplinary potential of theory-based mHealth tools (22).

However, not all studies reported superior results for mobile interventions. Zahid et al. (18) observed better toothbrushing frequency in the control group that received educational lectures, although a slight improvement in brushing technique was noted in the intervention group. Aljafari et al. (12) also found no significant difference in brushing frequency between children receiving game-based education and those who received traditional one-on-one instruction, although adherence to twice-daily brushing was maintained in both groups. These findings suggest that while digital tools can be effective, their impact may not always surpass conventional methods, particularly when baseline engagement or parental involvement is already high. This raises the possibility that mHealth tools may function best as adjuncts rather than replacements for traditional education, especially in populations where parental modelling and supervision strongly influence children's behaviours (22). The novelty-effect of apps may also diminish over time, reducing sustained engagement unless refreshed content or adaptive features are integrated (23).

It is also important to consider age-related differences in digital engagement. Most studies targeting younger children (e.g., aged 3–7 years) reported high levels of motivation and behavioural change, possibly due to the novelty and interactivity of mobile applications. Conversely, adolescents showed more mixed results, as seen in the study by Lopes dos Santos et al. (17), where clinical outcomes improved in the intervention group but did not reach statistical significance. Developmental psychology suggests that younger children benefit more from play-based learning strategies, whereas adolescents may require autonomy-supportive approaches, peer validation, or social media integration to sustain interest (24–26). This indicates the need for age-tailored app designs that reflect developmental and motivational differences.

Parental involvement also emerged as a crucial factor influencing outcomes. Several studies indirectly highlighted the role of parents in reinforcing behavioural messages delivered through mobile apps. Given the established association between maternal self-efficacy and children's oral health, future interventions may benefit from targeting both children and their caregivers simultaneously to maximize impact. Interventions that explicitly incorporate parental monitoring, feedback loops, or joint child–parent challenges are likely to improve adherence and oral health outcomes, as supported by evidence linking caregiver engagement with sustained behaviour change in pediatric dentistry (27).

Overall, the findings from this review support the integration of mobile-based and gamified interventions into pediatric oral health promotion strategies. These tools can effectively improve knowledge, increase engagement, and, in many cases, lead to measurable improvements in clinical outcomes. Nevertheless, the variability in study designs, sample sizes, follow-up durations, and outcome measures calls for more standardized research to establish generalizable conclusions. Future studies should consider incorporating behavioural change theories, ensuring parental engagement, and using validated outcome measures to better assess the long-term effectiveness of mobile health interventions in children's oral health. Furthermore, as digital health technologies evolve, future research should evaluate emerging innovations such as AI-driven personalization, integration with smart toothbrushes, and real-time feedback systems. These advancements could enhance scalability and long-term adherence but also raise important considerations regarding digital literacy, socioeconomic disparities, and data privacy (27, 28).

However, the review also has several limitations that warrant consideration. The most notable is the considerable heterogeneity among included studies, with differences in intervention formats, study populations, and outcome measures making direct comparisons difficult and precluding meta-analysis. This issue has been observed in other mHealth reviews as well, where variability in study designs limits the generalisability of conclusions (22, 23). In addition, most studies had relatively short follow-up periods, often limited to a few weeks or months, which restricts understanding of the long-term sustainability of digital interventions. Small sample sizes and potential risks of bias, including lack of randomisation or blinding in some trials, further limit the robustness of the evidence base. Another weakness lies in the lack of standardised outcome measures, with different plaque indices, oral hygiene knowledge questionnaires, and definitions of brushing adherence used across studies, which complicates meaningful synthesis of findings. Publication bias is also a concern, as positive outcomes of digital health interventions are more likely to be reported, potentially inflating their perceived effectiveness (20). Moreover, few studies conducted cost-effectiveness analyses, an essential consideration for scaling interventions into community or school-based programs (27). Finally, equity and accessibility remain important challenges, since the reviewed studies largely involved populations with ready access to smartphones and digital literacy, thereby excluding socioeconomically disadvantaged groups who may have the greatest need for scalable oral health interventions (28).

## Conclusion

This systematic review highlights the potential of mobile-based applications, particularly those incorporating game-based and interactive features, to improve oral health knowledge, hygiene behaviours, and clinical outcomes such as plaque scores and gingival health in children and adolescents. While several studies demonstrated significant improvements, the overall effectiveness of these interventions varied depending on factors such as age group, type of application, level of parental involvement, and study design.

Mobile health technologies represent a promising supplement to conventional oral health education, offering engaging, accessible, and scalable tools to promote behaviour change. However, further high-quality randomized controlled trials with standardized outcome measures and longer follow-up periods are needed to establish their long-term effectiveness and to inform evidence-based implementation in public health and clinical settings.

## Data Availability

The original contributions presented in the study are included in the article/Supplementary Material, further inquiries can be directed to the corresponding author.
